# Clinical outcomes of allograft with locking compression plates for elderly four-part proximal humerus fractures

**DOI:** 10.1186/s13018-015-0258-9

**Published:** 2015-07-22

**Authors:** Hua Chen, Xinran Ji, Qun Zhang, Xiangdang Liang, Peifu Tang

**Affiliations:** The Department of Orthopaedic Surgery, The General Hospital of People’s Liberation Army (301 Hospital), 28 Fuxing Road, Wukesong, Beijing 100000 China

**Keywords:** Four-part proximal humeral fracture, Locking compression plates (LCPs), Fibular shaft, Anatomical allograft

## Abstract

**Background:**

The aim of this study is to explore the clinical outcomes of anatomical allograft or fibula shaft augmentation with locking compression plates (LCPs) in elderly patients with four-part proximal humeral fracture (PHF).

**Methods:**

A total of 22 elderly patients with four-part PHF underwent allograft augmentation with LCPs for treatment. Among them, 7 cases received anatomical allograft and 15 patients received fibula shaft. Constant-Murley score (CMS), the disability of the arm, shoulder and hand (DASH) score, and subjective ratings, radiographic imaging, range of motion (ROM), and complications were recorded as postoperative evaluations.

**Results:**

Although the ROM and strength were considerably limited compared with the normal side, there were no significant differences in pain and daily activity between the unaffected and affected sides at the last follow-up according to the CMS. Additionally, no significant differences were found in the subjective ratings and CMS and DASH scores between the patients augmented with fibular shaft and anatomical allograft. Among the 15 patients who received fibular shaft, one case developed avascular necrosis (AVN) and screw cutout, but satisfactory outcomes were obtained after removal of implant. Besides, varus displacement occurred in one case, the patient acquired good function without revision. There were no infection, bone nonunion, and hardware-related complications occurred in any case.

**Conclusions:**

Both anatomical allograft and fibula shaft with LCPs showed relatively good clinical outcomes for elderly patients with four-part PHF.

## Background

Proximal humeral fractures (PHFs) account for approximately 10 % of all fractures [1], and the incidence is increasing with age [2]. Most PHFs are low-energy osteoporotic injury occurred in the elderly and afflict two or three times as many women as men [3]. This fracture still remains a major challenge for surgeons worldwide [4]. It has been reported that approximately 80 to 90 % of patients with minimally displaced PHF can be managed by nonoperation [5]. Four-part PHF is the most severe type among PHFs according to the Neer classification [6]. Nonoperative treatment for four-part PHF often results in less favorable clinical and anatomical outcomes [7, 8]. Shoulder hemiarthroplasty (HA) is advocated for treatment of this fracture type by prevention of varus collapse, deformity, and avascular necrosis risk [9, 10]. However, the functions and outcome evaluations are still controversial [11].

Recently, locking compression plate (LCP) demonstrates satisfactory results for severely displaced PHF compared with conventional plate. The fixed-angle construct could improve the fracture stability and increase the resistance to pullout through the bone-plate interface with a single beam construct, especially useful in poor-quality cancellous bone of the proximal humerus. However, some complications, such as avascular necrosis (AVN), screw cutout, implant failure, plate impingement, head collapse, and infection, have been reported [12, 13].

Autologous bone grafting might be an alternative method for overcoming varus collapse. However, autologous bone grafting which harvested from the patients themselves has some complications, such as vascular or neurologic injuries, deep infections at the donor site, and deep hematoma formation, while the efficiency of allograft bone grafting with LCP was rarely reported.

In the present study, we evaluated the outcomes of elderly patients with fresh four-part PHF who underwent fibular shaft or anatomical allograft for restoration of medial strut with LCPs.

## Materials and methods

### Participants

This research was a retrospective study and was approved by the Ethics Committee of General Hospital of People’s Liberation Army (301 Hospital). Written informed consents were obtained from all enrolled patients.

Between January 2010 and December 2011, patients who met the following inclusion criteria were recruited: (1) the patients suffered from an acute four-part PHF with or without fracture dislocation and (2) patients’ fragments were either displaced more than 1.0 cm or angulated more than 45° and were preoperatively conformed by radiograph or computed tomography (CT) with three-dimensional (3D) reconstructions. Patients who had any previous history of shoulder surgery or chronic nonunion, and addiction of cigarettes and/or drugs were excluded. Those who later refused to participate or failed to cooperate with us in this trial were also excluded.

### Preparation of allograft for medial support

**Fig. 1:**
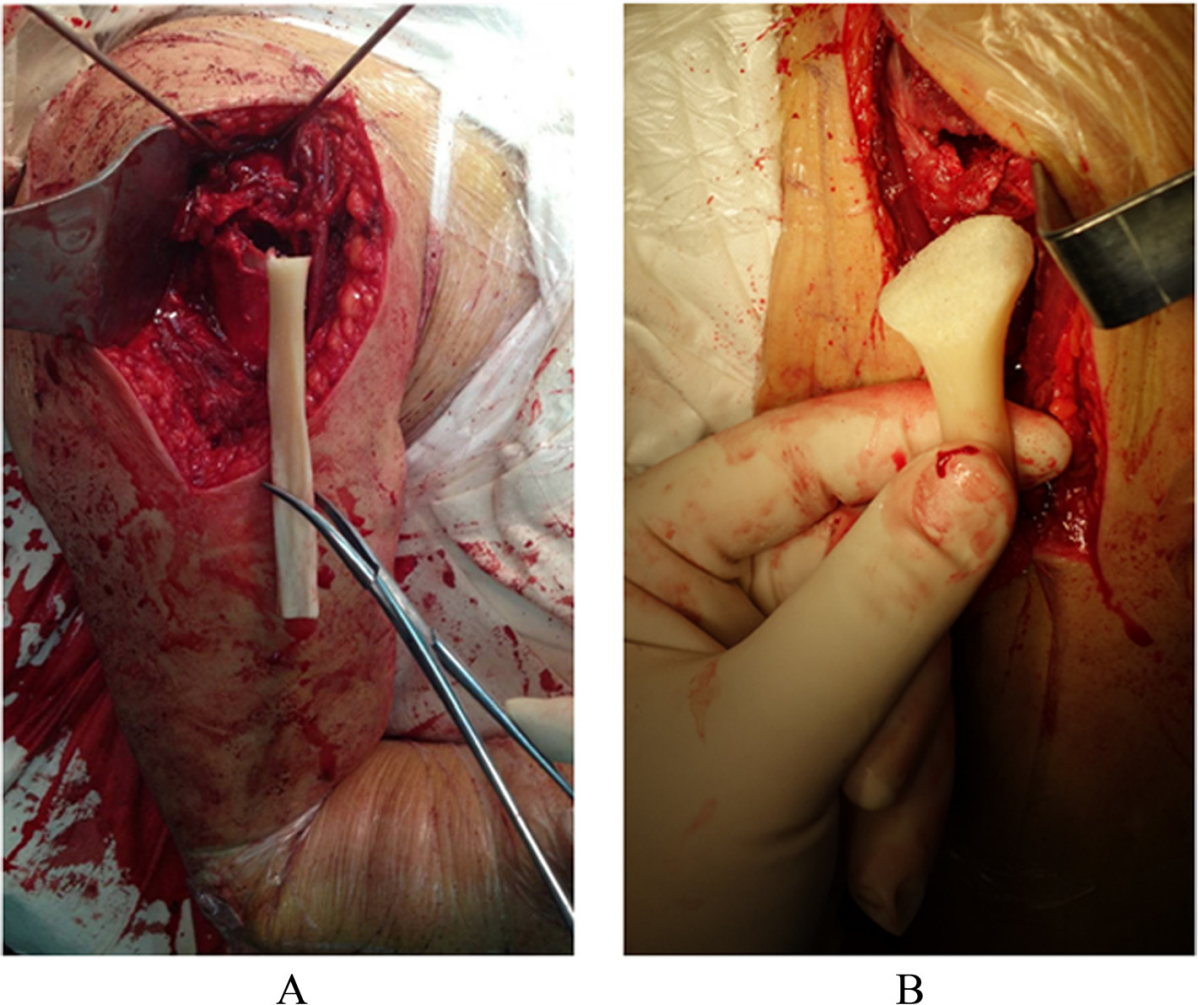
Two kinds of allografts for medial support. **a** Fibular allograft. **b** Anatomical allograft

There are two kinds of allografts for medial support: fibular allograft and anatomical allograft (Fig. 1). Fibular allograft was obtained from bone bank where it was cut into an appropriate length using a sagittal oscillating saw. Anatomical allograft was obtained from cadaveric donors. It was modified into a specific shape to fulfill the bone void according to the intramedullary geometry of the proximal humerus through computer virtual design with Pro-E software. All the strut allografts were stored at temperatures between −60 °C and −80 °C until use. It is important to emphasize that some blood-transmitted diseases in the allografts, e.g., acquired immune deficiency syndrome, hepatitis B and C, or syphilis, should be excluded.

### Operative technique

All surgical procedures were conducted by two of our well-experienced authors (Hua Chen and Peifu Tang). The choice for fibular shaft or anatomical allograft was made randomly. Briefly, the patients were placed in a beach chair position followed by general anesthesia and then received a standardized approach that was described previously [14]. Preoperative skin preparation was applied to the affected hemi-extremity. Approximately a 15-cm skin incision was made. The insertion of the deltoid muscle was one-half detached posteriorly subperiosteally. All patients received 1.5 g of cefuroxime preoperatively.

**Fig. 2:**
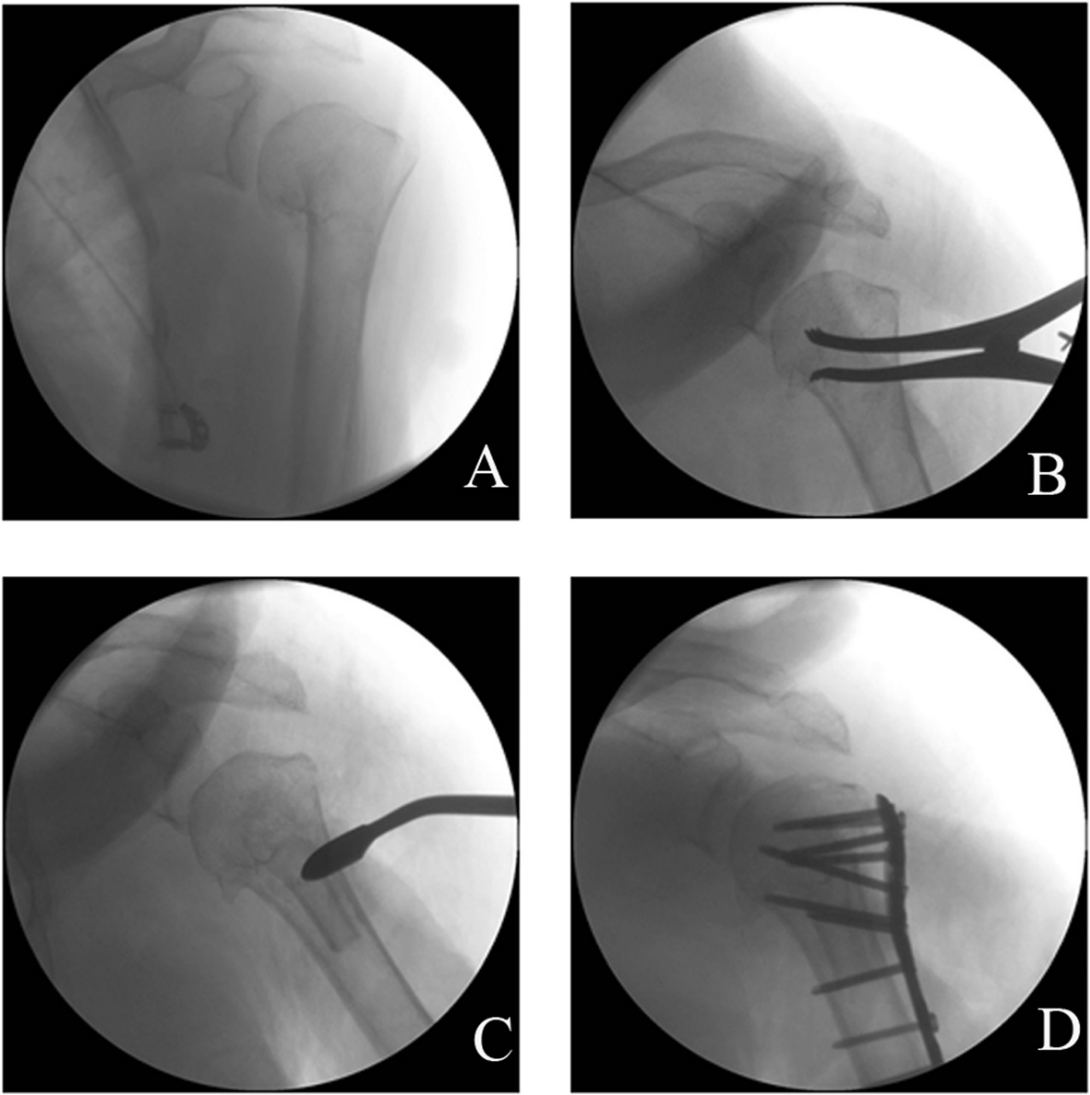
The humeral head and shaft are reduced with the help of laminar spreader under fluoroscopy and fixed by locking compression plates. **a** Before reduction. **b** Reduction with the help of laminar spreader. **c** Insertion of fibula. **d** Fixation by steel plate and screw

After the fracture sites were being exposed thoroughly, the long head of the biceps was identified and the configuration of the fracture was checked. Then, laminar spreader was put into intramedullary canal through lateral cortical window of tuberosity fracture sites with 30° of retroversion. The elbow was kept anteriorly. The humeral head and shaft were reduced with the help of the laminar spreader under (Fig. 2), especially when the medial calcar continuation and the normal neck shaft angle were restored. To prevent damaging the vascular supply to the humeral head, management of the articular segment was employed extracapsularly. Shaft traction was maintained by a surgical assistant. The intramedullary strut was inserted into the intramedullary canal distal to fracture site and was then driven back to the proximal humeral bone. Intramedullary strut allograft was pushed onto the medial calcar to support the humeral head for prevention of varus displacement and deformity of the humeral head. After that, the greater tuberosity fragment was reduced and sutured with No. 5 Ethibond sutures to maintain the reduction. The LCPs (Synthes, Switzerland) were placed between 5–10 mm lateral to the bicipital groove and 15–20 mm inferior to the vertex of the humerus head. Head-locking screws were placed in the subcortical bone, and the distal screws were placed in the shaft. The location of the screw tip was confirmed by an image intensifier. After careful irrigation, a negative suction drain was placed in the wound followed by layer closure.

### Postoperative rehabilitation

The shoulders were immobilized in a sling postoperatively. Patients received passive mobilization and pendulum exercises immediately. Additionally, physiotherapy was performed to all patients and gradually discontinued about 3 weeks. Both passive- and active-assisted exercises were permitted in a rehabilitation center at the second week after surgery. The forward elevation and abduction was limited to 100°, and external rotation was limited to 30°.

### Clinical and radiological evaluation

The subjective ratings of the outcome include excellent, good, fair, and poor. Functional outcomes were assessed using the disability of the arm, shoulder and hand (DASH) score [15] and Constant-Murley score (CMS) [16]. Bone mineral density (BMD) was assessed by dual-emission X-ray absorptiometry (DXA). Radiographs (standard AP position, axillary, and scapular Y views) were reviewed postoperatively. The radiographic follow-up periods were postoperatively 4, 8, and 13 weeks and then again 12, 24, and 48 months following surgery. However, implant should be removed when there were hardware-related symptoms. In addition, complications, such as varus displacement of humeral head, AVN, screw penetration, and infection, were also recorded.

### Comparison of functional outcomes

Functional outcomes such as pain, activity, range of motion, and strength of the fractured side and the normal side of patients between the two surgery groups were evaluated and compared.

### Statistical analysis

Continuous variables, presented as the mean and standard deviation (SD), were compared by the Student *t* test to detect the group differences. Qualitative data of groups was compared by the *χ*^2^ test. Statistical analysis was performed by SAS Statistical Software 9.1.3 (SAS Institute Inc., Cary, NC, USA). *P* value <0.05 was considered as significant difference.

## Results

### Patients’ characteristics

Finally, a total of 22 cases consisting of 9 males and 13 females were recruited in our study. The mean age was 67.2 ± 9.0 years (range, 52–84 years). The injury was mostly caused by falling from standing height (*n* = 17). The other six cases were injured from fracture dislocation (*n* = 4) and valgus-impacted fractures (*n* = 2). The mean length of the calcar segment was 6.4 ± 4.4 mm. The average medial hinge displacement was 13.4 ± 9.6 mm. The average dual BMD was −2.5 ± 0.5. The mean surgical time was 2.1 ± 0.8 h. The average intraoperative blood loss was 238 ± 83 ml. Seven cases received anatomical allograft and 15 cases received fibula shaft with LCPs for treatment of PHF (Table 1).

**Table 1:** Basic information for patients included in this study

Number	Gender	Age	Involvement	BMD	Injury cause	Length of calcar segment (mm)	Displacement of medial hinge (mm)	Displacement direction	Surgical time (h)	Blood loss (ml)
1	M	57	Rt	−2.6	FSH	4	13	Valgus-impacted	2.1	250
2	F	63	Rt	−2.9	FSH	6.6	5	Valgus-impacted	1.25	200
3	F	59	Lt	−2.2	FB	–	–	Dislocation	3.2	300
4	F	67	Lt	−3.2	FSH	3	12	–	1.5	100
5	M	66	Lt	−2.8	FSH	4	15	–	2	300
6	M	72	Rt	−3.1	FSH	–	–	Dislocation	3.1	500
7	F	73	Rt	−2.1	FSH	8	7	–	2	200
8	F	67	Rt	−2.9	FSH	8	10	–	2	300
9	F	66	Lt	−3.1	FSH	–	–	Dislocation	4	300
10	M	62	Rt	−1.9	TA	20	20	–	3	200
11	F	77	Lt	−2.8	FSH	5	5	–	1.25	150
12	M	77	Lt	−2.5	FSH	8	10	–	3	200
13	F	54	Rt	−2.4	F1M	8	3	–	1.75	200
14	M	52	Lt	−1.9	FSH	5	16	–	1.75	150
15	F	77	Lt	−3.1	FSH	–	–	Dislocation	2.75	300
16	M	71	Rt	−2.1	FSH	8	17	–	1.75	200
17	F	54	Lt	−2.3	F1M	4	14	–	1.25	200
18	M	84	Lt	−2.5	TA	12	25	–	2.5	300
19	F	79	Lt	−2.6	FSH	3	10	–	1.75	300
20	F	75	Lt	−1.6	FSH	5	16	–	1.25	200
21	F	65	Lt	−2.1	FSH	4	18	–	1.5	200
22	M	61	Rt	−1.6	FSH	4	16		1.5	200

*M* male, *F* female, *Rt* right, *Lt* left, *BMD* bone mineral density, *FSH* falling from standing height, *F1M* falling from 1 m high, *TA* traffic accident, FB falling from the running bicycle

### Clinical and radiologic outcomes

The mean follow-up period was 33.4 months (range, 24–48 months). No patient quitted during the whole follow-up period. The rating outcome was “excellent” in 19 cases and “good” in 3 cases (Table 2). The average CMS was 78.2 points (range, 66–90 points), and the mean DASH score was 8.1 points (range, 5.0–13.3).

**Table 2:** Clinical evaluations at the last follow up

Number	Allograft pattern	Follow-up (months)	Neck-shaft angle (degree)	Subjective evaluation	CMS	DASH	Complication	Notification
1	Fibular shaft	48	125	Excellent	86	5		
2	Fibular shaft	28	137	Excellent	72	9.2		
3	Fibular shaft	30	120	Excellent	80	10.8	AVN, screw cutout	Removal of implant
4	Fibular shaft	32	140	Excellent	80	6.7		
5	Fibular shaft	37	137	Excellent	79	6.7		
6	Anatomical allograft	42	150	Good	68	7.5		
7	Fibular shaft	45	135	Excellent	85	5.8		
8	Fibular shaft	40	135	Excellent	77	10.8		
9	Fibular shaft	39	120	Excellent	79	6.7		
10	Fibular shaft	35	135	Good	76	5.8		
11	Fibular shaft	25	120	Excellent	82	7.5		
12	Fibular shaft	27	140	Excellent	72	10		
13	Fibular shaft	30	135	Excellent	82	9.2		
14	Fibular shaft	38	90	Excellent	76	5.8	Varus displacement of humeral head	Observation
15	Anatomical allograft	31	120	Excellent	66	13.3		
16	Anatomical allograft	30	124	Excellent	79	9.2		
17	Anatomical allograft	36	134	Excellent	74	9.2		
18	Anatomical allograft	24	110	Good	82	10		
19	Anatomical allograft	27	120	Excellent	74	6.7		
20	Anatomical allograft	26	120	Excellent	85	9.2		
21	Fibular shaft	28	120	Excellent	90	5.8		
22	Fibular shaft	36	110	Excellent	76	6.7		

*CMS* Constant-Murley score, *DASH* the disability of the arm, shoulder and hand, *AVN* avascular necrosis

### Functional outcomes

Functional results at the last follow-up are shown in Table 3. Although there were no significant differences in pain (*P* = 0.7145) and activity (*P* = 0.6396) between the fractured side and the normal side, the range of motion (ROM) (*P* < 0.05) and strength (*P* < 0.05) were considerably limited compared with the normal side.

**Table 3:** Functional results at the last follow-up according to the CMS

Variable	Fractured side (*n* = 22)	Normal side (*n* = 22)	*P* value
Pain (0–15)	13.6 ± 2.8	13.9 ± 2.6	0.7145
Activity (0–20)	9.1 ± 1.9	8.8 ± 2.3	0.6396
ROM (0–40)			
Abduction	6.7 ± 1.5	9.3 ± 1.2*	0.0000
Anterior elevation	7.4 ± 1.3	8.9 ± 1.2*	0.0003
External rotation with elbow at the side	7.5 ± 1.5	9.7 ± 0.7*	0.0000
Internal rotation in abduction	7.7 ± 1.0	8.9 ± 0.9*	0.0001
Strength (0–25)	16.5 ± 3.4	23.9 ± 2.0*	0.0000

*CMS* Constant-Murley score, *ROM* range of motion

^*^ *P* < 0.01

### Comparisons of the clinical outcomes

Table 4 showed that no significant differences were found in subjective ratings (*P* = 0.163), CMS (*P* = 0.137), and DASH (*P* = 0.064) at the last follow-up between the patients augmented with fibular shaft and anatomical allograft. Also, there were no significant differences in patient subjective ratings (*P* = 0.727), CMS (*P* = 0.061), and DASH (*P* = 0.059) between the patients with shoulder dislocation and without dislocation.

**Table 4:** Comparison among clinical outcomes

		Excellent	Good	Fair	Poor	*P* value	CMS	*P* value	DASH	*P* value
Pattern	Fibular shaft (15)	14	1	–	–	0.163	79.47 ± 5.012	0.137	7.5 ± 1.967	0.064
	Anatomical allograft (7)	5	2	–	–		75.43 ± 7.020		9.3 ± 2.102	
Displacement	Dislocation (4)	3	1	–	–	0.727	73.25 ± 7.27	0.061	9.6 ± 3.052	0.059
	No dislocation (18)	16	2	–	–		79.28 ± 5.13		7.74 ± 1.83	

*CMS* Constant-Murley score, *DASH* the disability of the arm, shoulder and hand

**Fig. 3:**
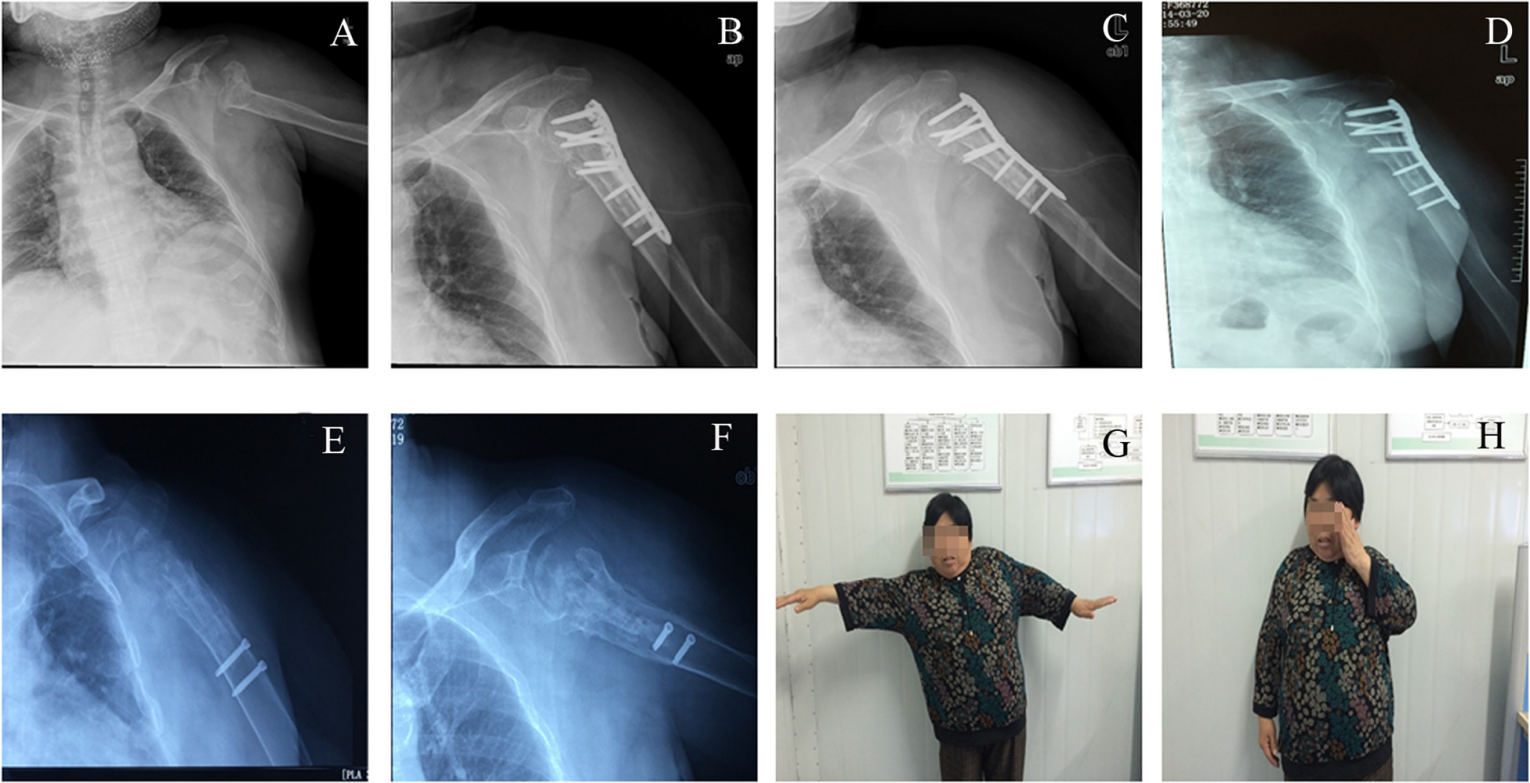
One case develops AVN and screw cutout but gets satisfactory outcomes after the implant is being removed. **a** X-ray film before surgery. **b** X-ray film after surgery. **c** X-ray film 3 months after surgery. **d** X-ray film 12 months after surgery. **e**, **f** X-ray film 30 months after surgery. **g**, **h** Function of patient’s upper arm

**Fig. 4:**
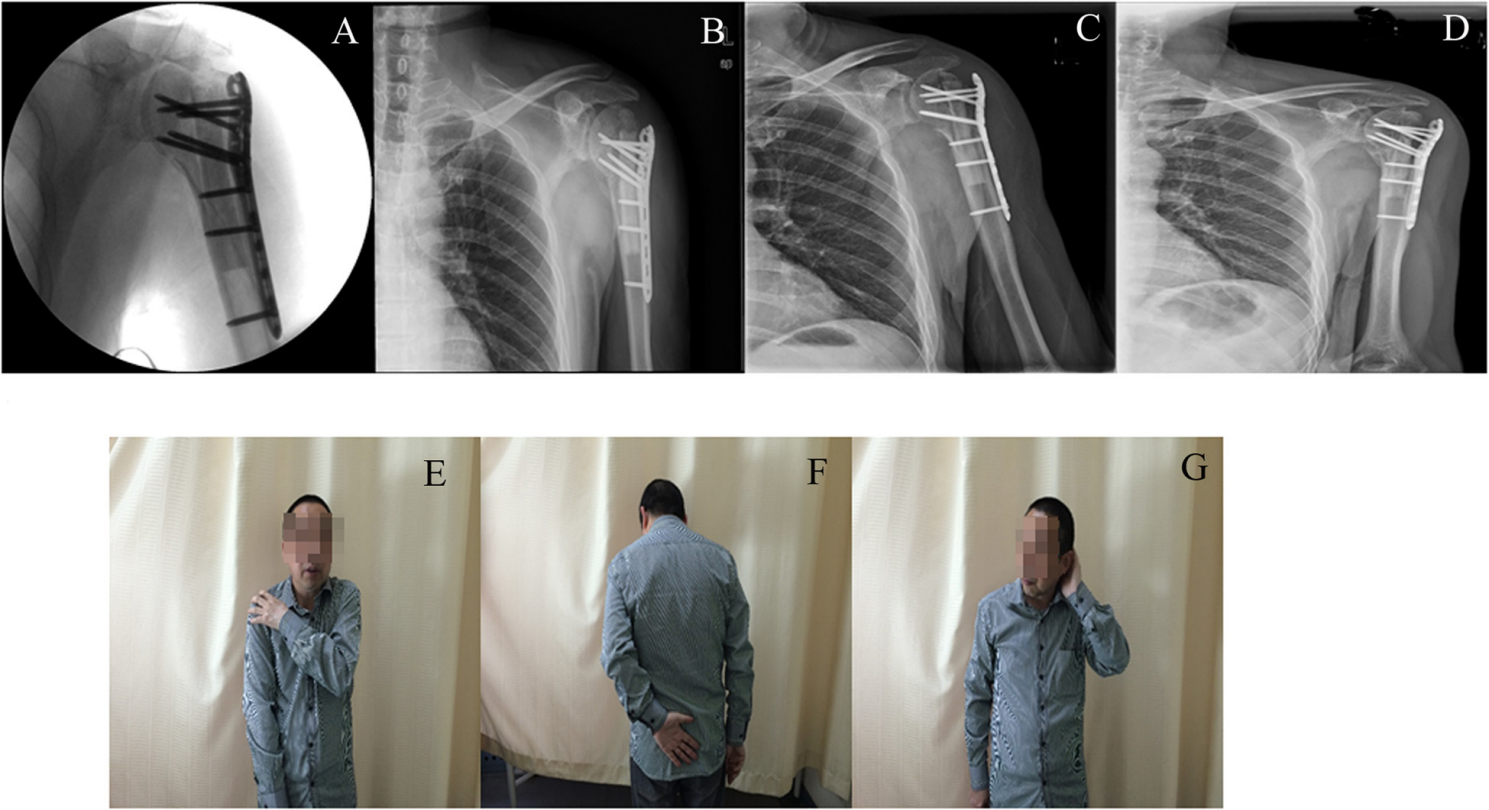
One case develops varus displacement but acquires good function without need of revision. **a** X-ray film before surgery. **b** X-ray film 3 days after surgery. **c** X-ray film 3 months after surgery. **d** X-ray film 38 months after surgery. **e-g** Function of patient’s upper arm

There was no infection or bone nonunion in all of the patients. The mean neck-shaft angle at the last follow-up was 126° ranging from 90° to 150° (data not shown). Among the 15 patients who received fibula shaft, one case developed AVN in the humeral head in combination with screw cutout. After the implant being removed, satisfactory outcomes were achieved (Fig. 3). In addition, one patient encountered varus displacement, but the patient acquired good function without the need of revision (Fig. 4).

## Discussion

PHF has been ranked as the third frequent fractures among elderly patients, followed by hip fractures and distal radius fractures [17], and has a strong correlation with osteoporosis [18]. Most of PHFs can be managed nonoperatively but with decreased shoulder fusion [19]. The four-part PHF represents about 3 % of all PHFs and is regarded as one of the most difficult PHFs to deal with [3]. In our series, we investigated the clinical outcomes of anatomical allograft or fibula shaft in treatment of LCPs in old people four-part PHF. The results showed that the efficacies of fibular shaft and anatomical allograft surgery strategies were similar, and both of them produced relatively satisfactory anatomical and functional outcomes.

The reconstruction of medial stability of the proximal humeral fracture sites seemed to promote fracture healing or revascularization of the humeral head, especially for patients with glenohumeral dislocation or medial comminution. In 1996, Walch et al. [20] firstly reported intramedullary strut grafting for treatment of the humerus neck nonunion. Peng et al. [21] found that adjuvant use of intramedullary strut allograft could significantly enhance bone union in elderly patients with three- and/or four-part fractures. Russo et al. [22] used triangular allogeneic grafts in 33 patients with sustained three- or four-part fractures; apart from one patient who encountered AVN, all fractures healed successfully. Lorich et al. [23] employed an endosteal cortical allograft strut in 38 patients with displaced PHF and found that this new technique reduced complications related to LCPs and improved clinical outcomes. Similarly, in our series, anatomical allograft or fibula shaft with LCPs was performed in elderly patients with four-part PHF. The results indicated that there were no significant differences in patient subjective ratings and CMS and DASH scores between the patients augmented with fibular shaft and anatomical allograft. Interestingly, the ROM and strength were considerably limited compared with the normal side in our study, which might be different from other studies. The possible reasons may be that the participants in our study were all elderly patients whose rehabilitation ability was limited. However, there were no significant differences in pain and daily activity between the unaffected and affected sides at the last follow-up according to the CMS. Although one case developed AVN and screw cutout, and another case developed varus displacement, both of them acquired good function at the last follow-up. Moreover, there were no infection, bone nonunion, and hardware-related complications in any case. All the above results suggested that medial stability could promote revascularization of the humeral head and allow bone fracture healing.

The similarly good clinical outcomes in our study might be related with some factors. Fibular allograft used as volumetric filling in the bone void formed after reduction of humeral neck-shaft angle could push the humeral head resistance to the force from the scapular fossa along with the screws, preventing the screw penetrating into the articular surface. Besides, this medial strut could prevent the varus placement of the head to diminish humeral head varus collapse and reduce the incidence of malunion. Anatomical medial strut with allograft bone has more potential to prevent humeral head varus displacement compared with the isolated fibula allograft. Anatomical allograft should be modified into a specific shape to fill the bone void according to the intramedullary geometry of the proximal humerus through computer virtual design with Pro-E software. This kind of structural allograft provides enough medial stability and allows the formation of osteogenic tissue across a fracture site along with the surface of the allograft followed by bone formation. In addition, faster fracture healing could minimize articular segment AVN or collapse. Anatomical allograft is a plane contacted with the humeral head, and the support position could be pushed to the inferior medial point. However, isolated fibula was just a point-to-point support of the humeral head, and the support point is just at the line of extension of the intramedullary canal direction.

## Conclusions

In conclusion, fibular shaft or anatomical allograft with LCPs produces similarly functional results and satisfactory results. The implantation promotes fracture healing without interfering blood supply to the humeral head.
